# Preparation and quality evaluation of sardines sauce rich in omega-3 using the combination of pineapple fruit extracts and fermentation times

**DOI:** 10.1007/s13197-020-04445-y

**Published:** 2020-04-22

**Authors:** H. Mahrus, Lalu Zulkifli, Dewa Ayu Citra Rasmi, Prapti Sedijani

**Affiliations:** 1 Biology Education Program, Education Faculty, University of Mataram, Jln. Majapahit No. 62, Mataram, Lombok-NTB 83125 Indonesia; 2 Graduate Program in Science Education, University of Mataram, Jln. Majapahit No. 62, Mataram, Lombok-NTB 83125 Indonesia

**Keywords:** Sardine, Pineapple, Bromelain, Fermentation, Physicochemical

## Abstract

A present study aimed at evaluating sardine sauce quality used a treatment combination of pineapple fruit extract, and fermentation times. It used a completely randomized design with a factorial pattern. The results showed a pineapple fruit extracts and fermentation times affected significantly on sardine sauce quality (P < 0.05). There was an interaction between pineapple fruit extract and fermentation times on sardine sauce quality. A pineapple fruit extract of 10% and fermentation times of 13 days produced sardine sauce best quality, with a protein content (17.38%), moisture (74.45%), omega-3 (19.68%), pH (5.23), taste value of 3.68, color of 4.52, and aroma of 2.99, respectively, but, consumers did not like it so much. It has passed a National Standard of Indonesia, which sets the minimum level of protein of 5%, and pH ranges from 5.0 to 6.0.

## Introduction

A Sardine (Sardinella lemuru) is one kind of marine fish that has a high content of omega-3 compared with other kinds of marine fish. Nowadays, it is used as the main food that is best for human health (Aung et al. 2018). Some marine fish species that contain high omega-3 are groupers and sardines. Sardine in the Lombok Strait waters of Indonesia consists of three variants containing an omega-3 of 23% (Mahrus et al. 2012). Some research findings in the past two decades have reported that omega-3 can prevent and cure coronary heart disease, maintain kidney health, diabetes, cancer, inflammation, and play a major part in central neuron system, brain, eyes and various other diseases (Calder 2012; Nabavi et al. 2015).

The production of the sardines in Lombok-Indonesia is abundant, but it does not get good handling, resulting in easy damage, and low price. One effort to increase the economic value and save the nutrition content of sardines is to use it as a sauce. Generally, in Indonesia, the raw materials to produce soy sauce from other countries with very expensive costs and taking a long time of the fermentation process. In addition to soybean sauce, it can use raw materials with better nutritional value, and low cost, namely from sardine fish originating from a sea. It means that a sauce can also be made from sardines fish with a short fermentation process by using the extracts of pineapple (*Ananas comosus*) fruit, which contains a bromelain enzyme.

Bromelain is a group of protein-digesting enzymes obtained commercially from the pineapple plant, can be used as an effectiveness health supplement to prevent cancer, diabetes and various cardiovascular diseases in the long run (Pavan et al. 2012). It means that the only enzyme, Bromelain, found in pineapple plants, has a function similar to omega-3 to prevent and cure cancer, heart disease, inflammation, help the digestive process, increases endurance, induces cell death apoptosis, heals wounds, etc. (Arshad et al. 2014; Rathnavelu et al. 2016). It can also be applied to hydrolyze fish proteins, degrade meat collagen, ferment fish sauce, and accelerate the process of protein hydrolysis (Le et al. 2015).

In contrast, applying a commercial bromelain enzyme, is very expensive, less stability, and it would be a big problem in producing a sauce in Indonesia. Therefore, pineapple is to be a good solution, because it is easy to find and a cheap price. Mature pineapple fruit is the best source of the bromelain enzyme and better stability at a high temperature (Arshad et al. 2014). Producing a sauce in a short time by using a bromelain enzyme from pineapple is to be a good alternative. References that focus on the studies publication related to the concentration combination of pineapple fruit extracts and fermentation times are still rare. Therefore, the present study is very important to initiate an effort of high-quality sauce production in the future by using wider resources, including the sardine and pineapple.

## Materials and methods

### Materials

The fresh sardines fish (Sardinella lemuru) as much as 10 kg, seven mature pineapple fruits, salt, spices of sauce in the form of cinnamon, ginger, bay leaves, lemongrass leaves, galangal, turmeric, and coriander, reagen biuret, benzoic acid, sodium acetate, K_2_CrO_4_, and AgNO_3_.

### Research methods

This research followed stages: (1) preparing the production of sardine sauce; (2) producing the sardine sauce; and (3) evaluating the quality of sardine sauce products.

1. Preparation the production of sardine sauce
  The experiment design used in this stage was an experimental laboratory using the randomized complete design with three replications. The treatment factor is different incubation times of L1 = 4, L2 = 7, L3 = 10, and L4 = 13 h. The raw material in preparing the production of sardine sauce consisted of 100 g sardine meat extract for each sample, pineapple fruit extracts 6% (w/w), 15% salt (w/w), and spices 1% (w/w).
  The first stage aimed at determining whether hydrolysis is successful or not based on hydrolyzate products. Parameters of hydrolyzate physicochemical were observed in this experiment including total nitrogen content, dissolved nitrogen, total dissolved solids, liquid volume, and viscosity. The physicochemical parameter value of hydrolyzate was determined with methods of the Association of Official Analytic Chemists (Andersen et al. 2018).
2. Producing the sardines sauce
  Methods used to produce sardines sauce is slightly modified from methods (Choi et al. 2018; Nakano et al. 2018). Flow chart of producing sardine sauce using bromelain enzyme is presented in Fig. 1. There are two stages of producing sardines sauce, namely: extraction of pineapple fruit into crude bromelain enzymes and hydrolysis of sardines meat to produce sardines sauce.

**Fig. 1:**
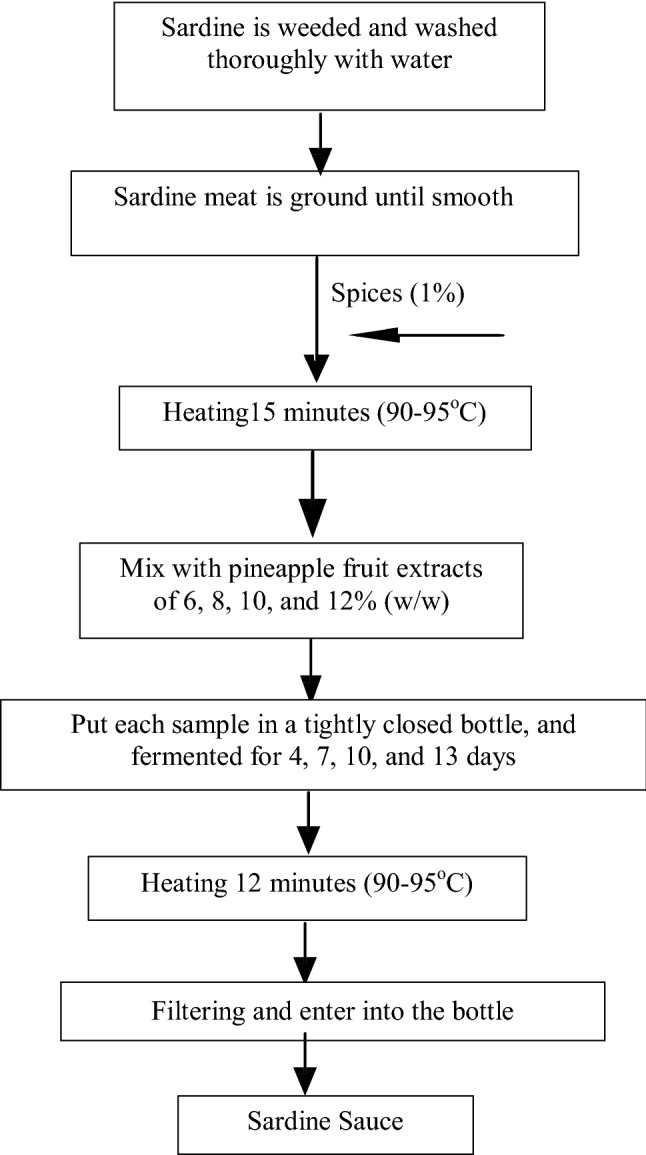
Flow chart of producing sardine sauce using bromelain enzyme
  The extraction methods of bromelain enzyme from mature pineapple fruit used slightly modified from methods (Ketnawa et al. 2011; Manzoor et al. 2016): five mature pineapple fruit was weighed, cleaned well-using water, and crushed into small pieces. Then, adding sodium acetate buffer into it, and the extract was homogenized and filtered by using a fine cloth. Filtrates obtained was centrifuged at 10,000 rpm for 10 min, and then the supernatants were collected, stored by adding 1.2 g of benzoic acid in it and used as crude bromelain enzyme.
3. Evaluating the quality of sardine sauce products

This experiment was done in a completely randomized design with two factors. The first factor was an added pineapple fruit extract containing bromelain enzyme with a concentration of EoLo = control (0%), E1 = 6% (w/w), E2 = 8% (w/w), E3 = 10% (w/w), and E4 = 12% (w/w) and the second factor was a fermentation duration of L1 = 4, L2 = 7, L3 = 10, and L4 = 13 days, respectively. Each treatment was replicated three times. Total protein, moisture, omega-3, pH, as well as sensory were measured.

The objective of this research is to evaluate the best quality product of sardine sauce by using a combination of different concentrations of pineapple fruit extracts and fermentation times. These studies were conducted at the Laboratory of Sciences Education and Laboratory of Analysis of Mataram University from April to November 2018.

Chemical parameters observed on stage 3 included protein content, moisture, omega-3, acidity (pH), and sensory evaluation score included flavor, odor, color, and overall pleasure. The control factors used in this study are without treatment, and analysis results of commercial fish sauce on the market as a comparison which covers four parameters, i.e. protein content, moisture, pH, and omega-3. Furthermore, the determination of protein content, salt content, and pH measurements were performed according to the methods published by the Association of Official Analytical Chemists (Andersen et al. 2018).

The extraction of the omega-3 content of the sardine sauce products was determined by using slightly modification methods to suit needed analytical requirements (Dincer et al. 2010; Opperman et al. 2011). It consisted of three stages: (1) the preparation of sample, (2) saponifications of sauce products, and (3) fractionization with urea, respectively.

At the preparation stage, sample analysis used sardine sauce products, and three commercial fish sauce purchased in the traditional market, respectively. The saponification stage of this study is to soap as much as 100 g of sauce for each sample using 80 g of NaOH solution in dilute alcohol (10 g of NaOH and 1.10 g of Ethylene Diamine Tetra Acetic Acid dissolved in 400 ml of distilled water and 400 ml of 96% ethanol) at room temperature for seven hours with constant stirring while flowing with nitrogen gas. Addition of 6 N HC1 solution into the saponification results until the pH of the solution reaches 1. The next is added n-hexane as much as 100 ml (several times) and evaporated with a rotary evaporator at a temperature of 30 °C.

Fractionization stage is conducted with the addition of 100 ml of hot urea solution at a temperature of 70 °C into 100 g of sauce saponification results. The ratio between urea with sauce liquid of 4:1, and 250 ml of methanol, and stirred the mixture until clear. The Urea and urea complex compounds are left overnight to crystallize at temperatures between − 36 to 36 °C, and then evaporation at a vacuum at room temperature. Adding concentrate with 0.1 ml HC1 of 125 ml and n-hexane of 125 ml, and separated the hexane layer. The lower layer was extracted again with 50 ml of n-hexane. The hexane phase mixture is vacuum evaporated at room temperature. A concentrate obtained is added with enough octyl gallate as a stabilizer. All concentrates were then concentrated to 1 ml before analyzing with gas chromatography (GC), and then to analyze two slightly different GC capillary selectivity columns. The determination of omega-3 fatty acid content used a GC method based on a heptadecanoic acid standard. It was determined as a percentage total of the omega-3 fatty acid content.

The sensory parameters observed included taste, color, aroma, and overall pleasure used 20 semi-trained panelists from instructors, undergraduate students, and technicians from the Department of Science Education, Faculty of the Education University of Mataram by using different materials properties. Sensory scores were evaluated using a nine-point hedonic scale with score one for most unacceptable and nine for most acceptable.

### Statistical analysis

All data from the experiment results were subjected to Analysis of Variance (ANOVA) and the differences between means were assessed by Tukey's test for statistical significance. The data were analyzed with SPSS (Ver. 21) software package. Tukey's honestly significant difference test (Tukey's HSD) test significance mean differences among samples. A significant difference between means was presented as means ± SD. A significant difference detected between treatments group (p < 0.05). The Tukey's test was used to measure certain more conservative differences using multiple comparisons. Besides, three commercial fish sauce samples were used to compare the quality of sardines sauce product based on values of the Indonesia National Standard (SNI 01-4271-1996) for the parameters of protein content and pH value.

## Results and discussion

### Preparing the production of a sauce

Some physicochemical parameters of hydrolyzate analyzed in preparation producing sauce were total nitrogen content, dissolved nitrogen, total dissolved solids, liquid volume, and the viscosity. This study reported the incubation times to increase, affected higher of total nitrogen value, dissolved nitrogen, total dissolved solids, liquid, volume, and viscosity. The highest levels of physicochemical parameters that took place in the hydrolysis times 10-h were total nitrogen (0.94%), dissolved nitrogen (0.75%), total dissolved solids (30.25%), fluid volume (14.10%), and viscosity (31.80%), respectively.

Based on the results of statistical tests using ANOVA, fermentation time has a significant effect on the physicochemical properties of hydrolyzates (p < 0.05). Furthermore, based on Tukey's test in Table 1, almost all treatments had a significant effect on the mean difference among of levels of total nitrogen, total dissolved solids, fluid volume, and viscosity (p < 0.05), except for the average levels between total nitrogen and dissolved nitrogen, and between viscosity and total dissolved solids. It means that the hydrolysis was successful.

**Table 1:** Tukey's multiple comparison test. Dependent variable: hydrolyzate products

	(I) physicochemical	(J) physicochemical	Mean difference (I–J)	Std. error	Sig	95% confidence interval	
						Lower bound	Upper bound
Tukey HSD	Total nitrogen	Dissolved nitrogen	0.18250	0.82699	0.999	− 2.3712	2.7362
		Total dissolved solids	− 27.99500^*^	0.82699	0.000	− 30.5487	− 25.4413
		Fluid volume	− 12.54250^*^	0.82699	0.000	− 15.0962	− 9.9888
		Viscosity	− 29.36250^*^	0.82699	0.000	− 31.9162	− 26.8088
	Dissolved nitrogen	Total nitrogen	− 0.18250	0.82699	0.999	− 2.7362	2.3712
		Total dissolved solids	− 28.17750^*^	0.82699	0.000	− 30.7312	− 25.6238
		Fluid volume	− 12.72500^*^	0.82699	0.000	− 15.2787	− 10.1713
		Viscosity	− 29.54500^*^	0.82699	0.000	− 32.0987	− 26.9913
	Total dissolved solids	Total nitrogen	27.99500^*^	0.82699	0.000	25.4413	30.5487
		Dissolved nitrogen	28.17750^*^	0.82699	0.000	25.6238	30.7312
		Fluid volume	15.45250^*^	0.82699	0.000	12.8988	18.0062
		Viscosity	− 1.36750	0.82699	0.489	− 3.9212	1.1862
	Fluid volume	Total nitrogen	12.54250^*^	0.82699	0.000	9.9888	15.0962
		Dissolved nitrogen	12.72500^*^	0.82699	0.000	10.1713	15.2787
		Total dissolved solids	− 15.45250^*^	0.82699	0.000	− 18.0062	− 12.8988
		Viscosity	− 16.82000^*^	0.82699	0.000	− 19.3737	− 14.2663
	Viscosity	Total nitrogen	29.36250^*^	0.82699	0.000	26.8088	31.9162
		Dissolved nitrogen	29.54500^*^	0.82699	0.000	26.9913	32.0987
		Total dissolved solids	1.36750	0.82699	0.489	− 1.1862	3.9212
		Fluid volume	16.82000^*^	0.82699	0.000	14.2663	19.3737

^*^The mean difference is significant at the 0.05 level

A method of enzymatic hydrolysis is currently suitable to convert the fish byproducts being value added-product, more marketable, and functional form. One of the benefits of bromelain is producing protein hydrolyzates. In making fish sauce, the protein is hydrolyzed by the enzyme bromelain into amino acids and peptides. Glutamine is one highly abundant amino acid found in most foodstuffs plays a role in a variety of biochemical functions, such as protein synthesis, and taste of sauce products. Choi et al. (2018) reported that glutamine accounted for about 40% of the total free amino acids, and there is an increased nitrogen content of anchovy sauces. A trend of increasing glutamic acid concentration with an increased nitrogen content of anchovy sauces affected its taste. Enzyme as a bio-catalysis has been successfully deployed for the multi-scale implementation because it is a key tool in biotechnology of seafood processing, cosmetic and pharmaceutical industries, freshness testing, etc. (Singh et al. 2013). This research is similar to results reported by Hall et al. (2010), the enzyme reaction also has the role important in increasing, decreasing, and stabilizing the physicochemical properties, although the bound enzyme concentration remains constant. Additionally, the enzyme also creates chemical reactions in the body, such as to perform very important tasks included building muscle, destroying toxins, and breaking down food particles during the digestions process.

Levels of hydrolysis success were determined through the total soluble solid total remaining. The total soluble solids content of the sauce treated with 6% of bromelain increased during the fermentation. The total soluble solids, measured using a refractometer, was used to estimate the degree of protein hydrolysis during the fermentation. Protein hydrolysis will release free amino acids and small (Hou et al. 2017). Total low solid residues indicate successfully bromelain enzyme in hydrolyzing fish meat. Using the right enzyme concentration to get a remaining solid and an enzyme concentration as low as possible is very important. Hall et al. (2010) reported that increasing the incubation time from 0 to 48 h would increase the value of nitrogen content (NC) and the degree of hydrolysis (DH). However, while the increasing incubation temperature from 30 to 60 °C produced an increase in NC, no significant difference was observed for DH.

### Evaluation of the quality of sardine sauce products

Overall, the treatment with the addition of different concentrations of pineapple fruit extracts and fermentation times affects the quality of sardine sauce products based on the chemical characteristic and sensory. The parameters of chemical observed included the average value of protein levels, moisture levels, omega-3 content, and pH, whereas sensory parameters consisted of taste, color, aroma, and overall pleasure. The best quality of sardine sauce product took place on the treatment E3L4 (pineapple fruit extract 10% with fermentation for 13 days) presented in Fig. 3. Aspects discussed followed are (1) chemical characteristic of sardine sauce product, (2) sensory of sardine sauce products, and (3) the comparative of quality for sardine sauce product and commercial sauce.

### Chemical characteristic of sardine sauce product

Chemical analysis of 16 sardine sauce product samples included protein content, moisture content, omega-3 content, and pH shows a difference compared with controls. Samples treated with pineapple fruit extract containing bromelain enzyme, and longer fermentation showed a significant increase in moisture content, otherwise, protein content, omega-3 content, and pH of sardine sauce product more decrease. It displayed that a higher pineapple fruit extract concentration and the longer fermentation time, the percentage of moisture content more increases (68.27 to 81.32%), while protein content from 18.98 to 16.18%, omega-3 of 29.92 to 16.85%, and pH ranges from 6.74 to 5.23, respectively. Factors causing a decrease and increase in some parameters of sardine sauce products are thought to be caused by enzymatic activity (Wang et al. 2017). It was carried out in mild conditions that can be easily controlled and allow to get products well-defined features. Additionally, the products produced depend on protein substrate, enzyme type, enzyme concentration, hydrolysis time, temperature, and pH, respectively. The most influential factor in this study was the concentration of pineapple extract containing a bromelain enzyme.

It has an important role in hydrolyzing protein into simpler compounds and tends to hydrolyze fish meat quickly and increase the quality sauce products. The fermentation process takes place well because the protein compounds hydrolyzed by the activity of enzymes found in the fish meat and pineapple fruit extract. Longer incubation times will give a longer chance for the enzyme to break down the substrate until a certain time limit to result in a constant reaction (Hall et al. 2010; Johnson and Goody 2011). In this study, there was a decrease in the levels of polyunsaturated fatty acids namely eicosapentaenoic acid and docosahexaenoic acid (EPA & DHA), more popularly called omega-3. This is caused by proteolysis activity gradually increased with the increase in enzyme concentration. It plays an important role as a catalyst to help speed up the hydrolysis process in fermentation for the fish sauce. Proteolysis activity gradually increases with higher enzyme concentration and hydrolysis time. Protein levels showed a tendency to decrease, and stable after the 10th day of fermentation. It caused the breakdown of fish proteins into simple compounds, namely amino acids, which are then converted into simpler compounds such as CO_2_, H_2_S, H_2_O, and other volatile compounds. It breaks fish proteins into smaller nitrogen components coming from enzymes present, either in the fish tissue, pineapple fruit extract, or microorganisms. Next, it hydrolyzes and oxidizes omega-3 during the fermentation process, so omega content decreases. The presence of water, the enzyme activity, and microbes can cause hydrolysis.

Unlike the case with moisture levels during fermentation tends to increase, which is between 67.82 to 816.32%. Proteolysis activity such as bromelain enzyme damage to protein causing the bound water to be free, and then increase the moisture content. Overall, enzymatic activity during the fermentation process will impact on the quality of sauce products.

Based on a statistical test using ANOVA presented in Table 2, pineapple fruit extract and fermentation times had a significantly different effect on protein content, moisture content, omega-3 content, and pH (p < 0.05). A Tukey test is carried out to find a significantly different sample. The results showed that overall there is a significant difference's effect among the treatments' group to mean value of protein content, moisture, omega-3, and pH (p < 0.05). Moreover, the interaction of the use of bromelain enzyme concentrations and fermentation times significantly affected on protein content, moisture, omega-3, pH, and organoleptic value of sardine fish sauce (P < 0.05). It means that their interactions impacted the quality of sardine sauce products.

**Table 2:** Mean difference using Tukey's multiple comparison tests. Dependent variable: sardine sauce quality

	(I) Chemical variables	(J) Chemical variables	Mean difference (I–J)	Std. error	Sig	95% confidence interval	
						Lower bound	Upper bound
Tukey HSD	Moisture content (%)	Omega-3 content (%)	50.10938^*^	1.31660	0.000	46.6349	53.5838
		pH	66.84250^*^	1.31660	0.000	63.3680	70.3170
		Protein content (%)	57.03770^*^	1.26356	0.000	53.7032	60.3722
	Omega-3 content (%)	Moisture content (%)	− 50.10938^*^	1.31660	0.000	− 53.5838	− 46.6349
		pH	16.73313^*^	1.31660	0.000	13.2587	20.2076
		Protein content (%)	6.92832^*^	1.26356	0.000	3.5938	10.2628
	pH	Moisture content (%)	− 66.84250^*^	1.31660	0.000	− 70.3170	− 63.3680
		Omega-3 content (%)	− 16.73313^*^	1.31660	0.000	− 20.2076	− 13.2587
		Protein content (%)	− 9.80480^*^	1.26356	0.000	− 13.1393	− 6.4703
	Protein content (%)	Moisture content (%)	− 57.03770^*^	1.26356	0.000	− 60.3722	− 53.7032
		Omega-3 content (%)	− 6.92832^*^	1.26356	0.000	− 10.2628	− 3.5938
		pH	9.80480^*^	1.26356	0.000	6.4703	13.1393

*The mean difference is significant at the 0.05 level

This result is almost the same as previous studies reported by researchers (Bentis et al. 2005; Le et al. 2015; Wang et al. 2017). Bentis et al. (2005) reported that moisture, protein, and lipid content had an important effect on the formation and the quality of the final product, such as fish sauce. The addition of commercial proteases (bromelain) could be helpful for the liquefaction of fish and cleavage of peptide bonds that occur during fish sauce production. It causes to speed up the production process and finally increases protein amount (Le et al. 2015). The results of this study were clarified more details by Wang et al. (2017), fermented fish products for six weeks can cause a gradual decrease of pH value and change the protein composition of Suanyu, a traditional fermented fish product of China, throughout a fermentation. Overall, changes in protein composition by intense proteolysis in Suanyu brought numerous small peptides and free amino acids, which contribute to the taste and flavor of Suanyu.

The previously similar studies reported protein content and moisture content on the fermented fish that is 71.25%, and 26.81%, respectively (Koffi-Nevry et al. 2011). Another research found 77.3% moisture, 18.2% protein on yellowfin tuna roe, as a raw material for one of the most valuable food products from fishery sources (Lee et al. 2016).

The parameters commonly used to determine the quality of fish sauce are levels of protein and pH. The standard pH and protein content according to the Indonesian National Standard ranged from five to six and at least 5%, respectively. This study showed that the best sardine sauce product is produced by the combination of concentration treatment of pineapple extract of 10%, and fermentation times of 13 days (E3L4), with the results of pH 5.23 and protein levels of 19.382%. This product means that a sardine sauce with protein levels of 19.38% and a pH of 5.23 can be free from the contaminated pathogenic microbe (Mohamed et al. 2012).

In contrast, fish sauce products in Malaysia called budu, the best fish sauce and the most popular in Malaysia, has a much lower of pH from 4.50 to 4.92 (Mohamed et al. 2012), and 14th-day fermented anchovy sauce with a pH of 4.47 to 4.89 (Choi et al. 2018) compared to these results (5.23 to 6.74). Fish sauce products with a pH of 6.52–6.74 should not be stored for a long time because it will damage the aroma and taste (Poll et al. 2003). The decrease of pH during the fermentation is thought to caused by the increase of the formation of amino acids and polypeptides because of the protein breakdown reaction (Le et al. 2015). The similar results also reported by Ghosh and Chattopadhyay (2011), there is an increasing trend of acidity level, i.e., a decrease in the pH value in making idli better at different fermentation periods. Besides, changes in pH are caused by acid production by bacterial activity as a result of the biochemical changes that occur during the fermentation process (Hou et al. 2017). The use of microorganisms can not only break down proteins into peptides and free amino acids but can also remove hyper-allergic of fish sauce This is mainly associated with the producing lactic acid which lowers the pH, for example, all volatile organic acids short-chain affects the acidity. However, it was not observed in this study.

The importance of a combination of fruit extract and fermentation long, establishing frameworks specifically for sardine sauce products, fermentation-enabled wellness foods, and functional fermented foods, are highlighted. The innovation of this study is to produce the best quality of sardine sauce according to the National Standard of Indonesia, especially for fish sauce. Based on these research findings, it is possible to provide opportunities for fish sauce industry applications aiming at increasing the health benefits of functional sardine sauce products.

### Sensory of sardines sauce products

The method used in testing sensory for sardine sauce in this research is the scoring test covering taste, color, aroma, and acceptance (overall preferences). All the treatments were evaluated by serving 20 semi-trained panelists from instructors, undergraduate students, and technicians from the Department of Science Education, Faculty of Education University of Mataram, Indonesia. The panelists were asked to evaluate the likability of color, odor, taste, and overall for each sample using a nine-point hedonic scale from one (dislike extremely) to nine (like extremely).

**Fig. 2:**
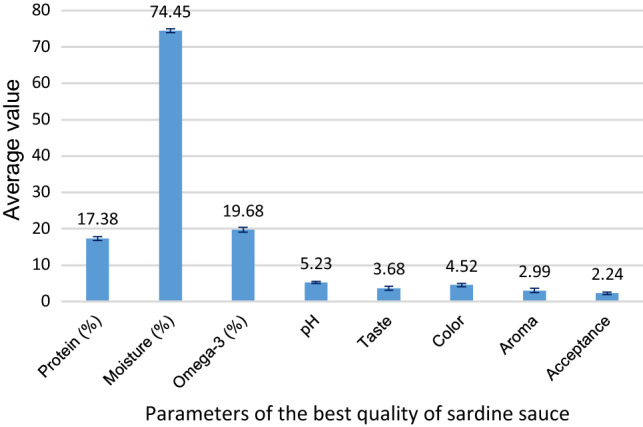
The average value of parameters of the best quality of sardine sauce

Based on the results of data analysis using ANOVA in Table 3, there was significant mean difference between sensory parameter value (p > 0.05). It means that the treatment combination of pineapple fruit extracts and fermentation times had a significantly different effect on the sensory parameter value. The most quality sardine sauce was prepared from E3L4 treatment with the ratio of 10% concentration of pineapple fruit extracts and 13th-days fermentation times with average value for a taste of 3.68, the color of 4.52, and aroma of 2.99, respectively. Figure 2 shows the value of the best sardine sauce quality parameters, both chemical and organoleptic. The great score for acceptance (overall pleasure) is 2.56 took place on treatment (E1L2) with pineapple fruit extract 6% and fermentation for seven days. The overall organoleptic value of fish sauce made with E1L2 treatment is the most accepted by consumers with a taste value of 2.3, a color value of 3.35, and an aroma value of 2.94.

**Table 3:** Mean difference using Tukey's multiple comparison tests. Dependent variable: sardine sauce quality

	(I) Sensory variables	(J) Sensory variables	Mean difference (I–J)	Std. error	Sig	95% confidence interval	
						Lower bound	Upper bound
Tukey HSD	Taste	Color	− 0.71938^*^	0.17685	0.001	− 1.1867	− 0.2520
		Aroma	0.10375	0.17685	0.936	− 0.3636	0.5711
		Acceptance	0.46625	0.17685	0.051	− 0.0011	0.9336
	Color	Taste	0.71938^*^	0.17685	0.001	0.2520	1.1867
		Aroma	0.82313^*^	0.17685	0.000	0.3558	1.2905
		Acceptance	1.18563^*^	0.17685	0.000	0.7183	1.6530
	Aroma	Taste	− 0.10375	0.17685	0.936	− 0.5711	0.3636
		Color	− 0.82313^*^	0.17685	0.000	− 1.2905	− 0.3558
		Acceptance	0.36250	0.17685	0.182	− 0.1048	0.8298
	Acceptance	Taste	− 0.46625	0.17685	0.051	− 0.9336	0.0011
		Color	− 1.18563^*^	0.17685	0.000	− 1.6530	− 0.7183
		Aroma	− 0.36250	0.17685	0.182	− 0.8298	0.1048

^*^The mean difference is significant at the 0.05 level

Sardines sauce produced from the combination of pineapple extract concentration of 10% and 13 days fermentation times is not liked by the panelist. The consumers do not prefer the sardine sauce products which has longer fermentation times. One of the causing factors is that the longer fermentation causes the pH of the sardine sauce to be more acidic. The other factors that influence consumer perceptions of food products are colors and sensory properties. Samborska et al. (2019) stated that both colors and sensory properties have a significant effect on the perception of consumers, especially in Osmo-dehydrated apples.

Consumer acceptability is a cumulative effect of tenderness, juiciness, taste, and color, as well as the appearance of the product. Ethanol treatment can decrease the taste of salty and the odor significantly (Liu et al. 2017). Nevertheless, it is not conducted in this research. Alcohol concentration was instrumental in improving vinegar-making technology from fruits, producing yeast starters, and strains of acetate bacteria for this production. Next, a concentration of ethanol is not a limiting factor for the growth of acetic bacteria (Lynch et al. 2019).

Treatment with higher pineapple fruit extracts and longer fermentation times has a stronger effect than the control treatment. Darker colors, slightly stronger scents and tastes from the sardines sauce is thought caused by the proteolytic enzyme activity during the fermentation. The aroma of chocolate, and the stronger aroma of fish, are likely to be a higher level of nitrogen compounds, probably produced during the production process. The longer fermentation times and more pineapple fruit extracts added, will cause a darker color. This result also showed that a longer fish sauce is stored, which will cause the color to be darker because of the absorbent increasing. Taste, aroma, and mouth stimulation generally will give rise to the emergence of a person’s feelings after eating the product. Therefore, numerical preference only is not enough to evaluate it. The numerical level of preference in this study was greatly influenced by panelists, even though many factors contributing to such as materials, methods, fermentation length (Gutiérrez-Salomón et al. 2014). Nevertheless, the overall results of this study were lower in quality compared to Thai fish sauce and Malaysia (Mohamed et al. 2012).

Furthermore, they reported that fermentation contributes to increasing the shelf life and sensory properties of food, especially the taste and the aroma. This result was similar to the previous study on wine product reported by Rodrigues et al. (2017), participants’ mood before tasting the wines had a strong effect on consecutive product-evoked emotion ratings, but the only weak effect on preference ratings. They also stated that although emotions and preferences are correlated, the measurement of emotions can deliver additional information over preference. The other studies reported that food-evoked emotions could add to preference ratings in explaining choice behavior and provides additional information to preference food products (Danner et al. 2016; Delime et al. 2020). They suggested that collecting emotion responses to detect changes in the emotional profile of products is very important. But, this study did not observe panelist's emotions. Rodrigues et al. (2017) reported that sensory analyses included minerality rating and free description performed by wine professionals under two conditions: orthonasal olfaction alone and global tasting. Chemical characterization included analysis of major and minor volatile compounds, volatile sulfur compounds, mercaptans, metals, anions, and cations.

### The comparative of quality for sardine sauce product and commercial sauce

**Fig. 3:**
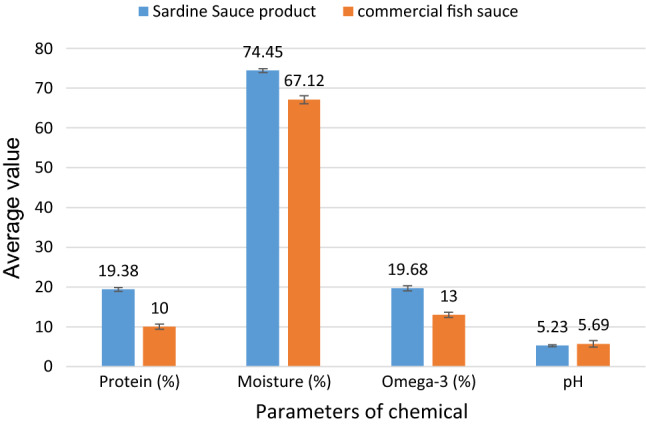
Quality comparative between sardine sauce product and commercial fish sauce using chemical parameters

The study used a commercial anchovy fish from a traditional market of Lombok island-Indonesia as a comparison in this study. It has a pH of 5.69, levels of protein 10%, and omega-3 by 13%. The quality of this commercial fish sauce product is lower than this study, as shown in Fig. 3. The levels of omega-3 in commercial anchovy sauce products are much lower with the levels of omega-3, belong to a sardine in Lombok strait waters of 23% (Mahrus et al. 2012). The content of omega-3 found of the best sardine sauce products in this research was high enough, which is 19.68%. The result of this study is similar to the previous study of fish sauce containing high omega-3 content reported by Dincer et al. (2010).

An advantage of sardine sauce products in the present study compared to commercial fish sauce on the market is having the highest omega-3 content that is very beneficial for human health (Siscovick et al. 2017). Therefore, Siscovick et al. (2017), from the American Heart Association, have recommended that the use of omega-3 for the prevention of cardiovascular heart disease (CHD) in individuals with previous CHD and heart failure patients. It reported that CVD was the most common underlying cause of death in the world in 2013. Heart failure is still a major health problem in developing countries, and its incidence is always increasing each year (Mendes-Ribeiro et al. 2009).

Besides, an ecological study in the last three decades in China reported there was a significant correlation between the monthly consumption of fish sauce and the mortality of gastric cancer. There was also evidence that a positive correlation between gastric cancer mortality and fish sauce favored by Fujian residents (Cai et al. 2000). Therefore, by consuming a sardine sauce, it is expected to be a solution to prevent heart failure incident and gastric cancer according to world health experts in this last decade reported that omega-3 s can prevent and cure coronary heart disease, diabetes, cancer, and play an important role in the nervous system, brain, and eyes (Calder 2012).

## Conclusion

It indicates that the treatment mixing the concentration of the pineapple fruit extract of 10% and the fermentation times of 13 days (E3L4) produced the best quality of the sardine sauce with the main properties as follows: protein level of 17.38%, moisture content of 74.45%, omega-3 content of 19.68%, pH of 5.23, taste value of 3.68, color of 4.52, and aroma of 2.99, respectively. These values were passed the Indonesian National Standard (SNI 01-4271-1996), which sets the level of minimum protein of 5%, pH 5.0 to 6.0, darker color, slightly stronger scent, and taste. The quality of these sardine sauce products is also better than that of the fish sauce available in the traditional market in Lombok Indonesia. Therefore, the methods of the preparation and the evaluation of the producing sardine sauce were appropriate to produce quality sardines sauce products.
